# Uterine artery embolisation for adenomyosis in women who failed prior endometrial ablation

**DOI:** 10.1186/s42155-024-00471-5

**Published:** 2024-07-27

**Authors:** Eisen Liang, Razeen Parvez, Sylvia Ng, Bevan Brown

**Affiliations:** 1 Sydney Fibroid Clinic, Sydney, Australia; 2 Gosford Hospital, Gosford, NSW Australia

## Purpose

To report the effectiveness of uterine artery embolisation (UAE) in treating adenomyosis in women who failed prior endometrial ablation (EA).

## Introduction

Endometrial ablation (EA) is a minimally invasive treatment for heavy menstrual bleeding (HMB). Patient satisfaction rates for EA are around 80–90%; however, about 10–20% of women require additional intervention (re-ablation or hysterectomy) due to persistent bleeding or pain [1]. Women with adenomyosis are more likely to fail EA [2]. Those with unsatisfactory outcomes from EA may be offered hysterectomy as their only remaining treatment option. Case series and meta-analyses have demonstrated that UAE is effective in alleviating adenomyosis-related HMB and dysmenorrhea [3, 4]. However, the effectiveness of UAE in treating women who failed prior EA has not been previously reported. This is a retrospective cohort study of the outcome of UAE for adenomyosis in women who failed previous EA.

## Materials and methods

This study was approved by the institutional Human Research Ethics Committee. Informed consent was obtained from each participant. Women presenting to our clinic with significant dysmenorrhea and/or HMB following unsatisfactory endometrial ablation were offered UAE as an alternative to hysterectomy. Medical records of women who had UAE for adenomyosis at our institution between January 2017 and March 2022 were reviewed to identify those who had EA prior to UAE. All women had pre-UAE MRI to confirm the presence of adenomyosis, diagnosed based on previously published criteria: junctional zone thickness of ≥ 12 mm or > 40% of myometrial thickness, or the presence of T2 hyperintense cysts/foci/fissuring [5]. All UAE procedures were performed with non-spherical polyvinyl alcohol (nsPVA) particles as previously described [4], and with starting nsPVA size at 180–300 micron (Cook 200) or 150–250 micron (Boston Scientific) as suggested by the 1-2-3 Protocol [6]. To evaluate the clinical outcome, a 2-part online survey was sent to women via email link to complete at home. Part 1 inquired about symptoms, menopausal status, overall satisfaction, and requirement for further intervention (Appendix 1). Women who had heavy menstrual bleeding prior to UAE were asked about their periods at the time of the audit. Overall success rate of UAE was assessed by asking women if they were “Very Satisfied,” “Satisfied,” “Not sure,” “Not Satisfied,” or “Very Unsatisfied” about the outcome. Only women who rated “Very Satisfied” or “Satisfied” were regarded as overall successful. Part 2 consisted of the validated Uterine Fibroid Symptom and Quality of Life Survey (UFSQoL) [7]. The following parameters before UAE and at follow-up were recorded and compared: dysmenorrhea visual analogue scale (VAS) pain score, number of days with dysmenorrhea, symptom score, and QoL score (using UFSQoL). Uterine volume and junctional zone thickness at baseline MRI and 6 months follow-up were compared. Significance of changes before and after treatment was analyzed using T-tests.

## Results

**Fig. 1:**
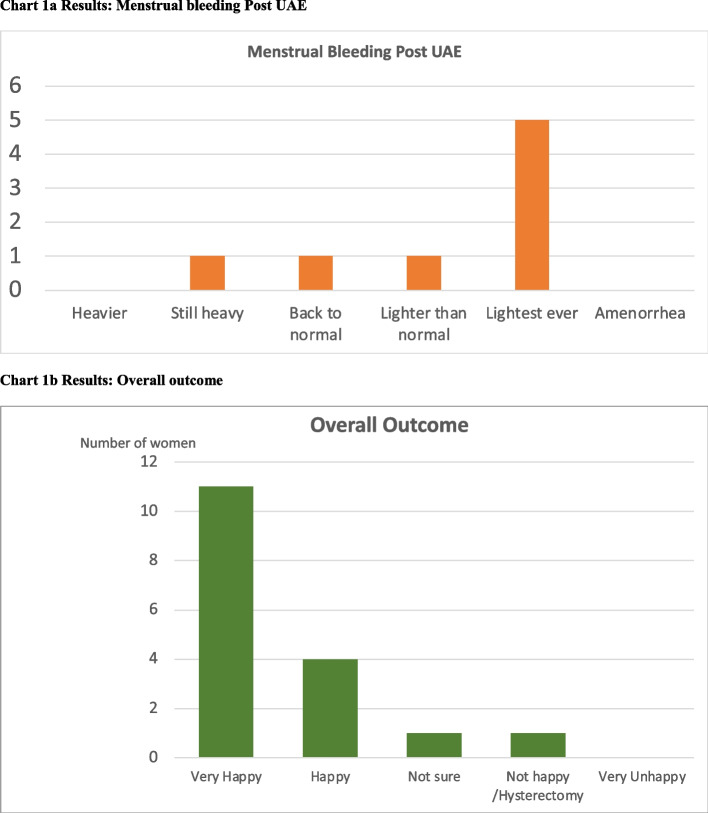
**a** Results: Menstrual bleeding Post UAE. **b** Results: Overall outcome

**Fig. 2:**
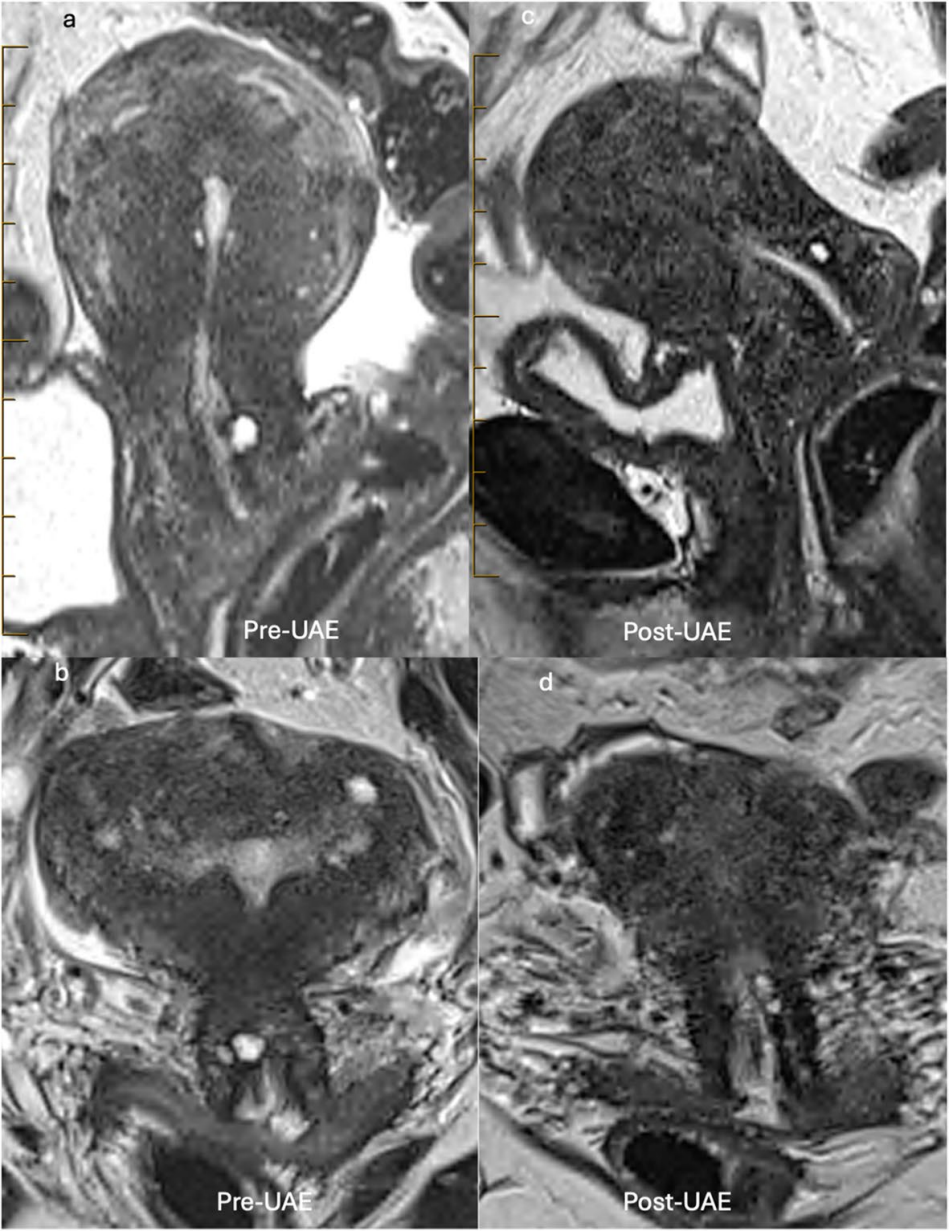
MRI of a 50-year-old woman who had endometrial ablation 10 months ago but was still suffering from heavy menstrual bleeding and severe dysmenorrhea. Following UAE, she had regular periods with lightest-ever bleeding and a reduction of pain score from 10 to 1. Sagittal T2 MRI pre- and post-embolisation demonstrated a reduction of uterine volume from 210 to 97 mL, reduction of junctional zone from 21 to 14 mm, less bulging globular appearance, and marked reduction of myometrial cystic spaces (ectopic endometrial tissue)

Between January 2017 and March 2022, UAE procedures were performed in 270 women for adenomyosis, some of whom also had fibroids. Eighteen women were identified with prior ablation failure (see Table 1): 15 with pure adenomyosis and 3 with coexisting fibroids. One woman was lost to follow-up; the outcomes of 17/18 (94.4%) were available for analysis, at a mean follow-up of 1.6 years post-UAE (median 2, range 0.6–3 years). Significant reductions in pain score (VAS 6.29), number of days in pain (2.6 days), symptom score (32.1/100), and significant improvement in QoL (39/100) were noted (Table 2). For the 8 women who still had HMB post-ablation, 7 (87.5%) saw significant improvement. Overall, 15/17 patients (88.2%) were “Satisfied” or “Very Satisfied” with the outcome of the UAE procedure (Fig. 1). Only 1 (5.9%) woman required a hysterectomy. No other women required further intervention such as laparoscopy for residual pain/endometriosis. No women reported menopause. No immediate or long-term complications were noted in this audit. Thirteen women (76.5%) attended follow-up imaging (Fig. 2). Significant uterine volume reduction and JZ thickness reduction were noted (Table 2).

**Table 1:** Baseline parameters

Baseline parameters	Mean	Median	Range
Age at time of procedure (years)	42.8	42.5	34–53
Age at time of UAE	47.7	49	40–54
Time gap between Ablation and UAE (years)	5.2	3.5	0.4–14
No of patients with Residual HMB post ablation (%)	8 (47%)	-	-
VAS Pain score post ablation	8.1	9	0–10
Number of days in pain post ablation	2.7	2	0–10
MRI Uterine volume prior to UAE (cc)	189.7	178	62–427
MRI JZ thickness prior to UAE (mm)	19.1	20	12–25

**Table 2:** Results: changes against baseline

Reduction of mean VAS dysmenorrhea pain score	6.29, (*P* < 0.0001) From 8.1 (median 9, range 0–10) to 1.9 (median 1, range 0–5)
Reduction of number of days in dysmenorrhea	2.6 From 2.7 (median 2, range 10) to 0.1 (median 0, range 2)
Improvement of mean UFS symptom score	32.1, (*P* < 0.0001) From 51.4 (median 53.13, range 25–100) to 19.3 (median 12.5, range 0–78.1)
Improvement of mean UFS QoL score	39.0, (*P* < 0.0001) From 46.0 (median 56.0, range 2.6–80.2) to 84.9 (median 94.0, range 33.6–100)
Improvement in menorrhagia (*n* = 8) (BTN, LTN, LTE)	7 (87.5%)
Clinical success (Satisfied or Very Satisfied with outcome)	15/17 patients (88.2%)
Mean Uterine volume reduction (mL)	66.5(35.1%), (*P* < 0.001) From 189.7 (median 178, range 62–427) to 123.3 (median 90, range 36–242)
Mean JZ thickness reduction (mm)	3.9 (*P* < 0.003) From 17.9 (median 16, range 6 -24) to 14 (median 11, range 8 to 27)

The woman who failed UAE requiring subsequent hysterectomy had pure diffuse adenomyosis and scored the smallest uterine volume (62 mL) of the study cohort. Her angiography showed low uterine vascularity and bilateral small uterine arteries, which were pretreated with 100 µg of glycerol trinitrate on each side, prior to embolisation with nsPVA (Cook 200).

## Discussions

This study has shown that UAE can be an effective treatment for post-ablation adenomyosis, with significant improvement in pain and residual HMB. Overall patient satisfaction is 88.2%, and 94.1% were able to avoid a hysterectomy. The results from this post-ablation failure cohort compare favourably with previously reported overall UAE outcomes for adenomyosis [3, 4]. UAE as a non-targeted particle embolisation causes global ischemia of uterus. We postulate that normal myometrium can recover and remains viable due to numerous underlying dormant collateral vessels that can be recruited; abnormal tissue like adenomyosis does not have spare vessels to recruit and will undergo irreversible ischaemic infarction.

The question of whether EA is appropriate to treat adenomyosis should be raised. Depth of involvement of adenomyosis has been shown to be associated with endometrial ablation failure [8]. Deep adenomyosis (> 2.5 mm) is present in a significant number of women who underwent hysterectomy after failed endometrial ablation [2]. EA devices are designed to cause thermal destruction of 4–6 mm depth of tissue to the basalis level [9]. MRI diagnosis of adenomyosis requires junctional zone thickness of 12 mm or more [5]. Therefore, if the diagnosis of adenomyosis is established based on MRI criteria, there is a high chance that endometrial ablation might fail. We strongly argue that if adenomyosis is established on imaging, UAE should be offered to women as an alternative to hysterectomy, as many case series has demonstrated the safety and effectiveness of UAE for adenomyosis [3, 4]. Previous studies suggest that ablation either activates surviving endometrial tissue to penetrate the myometrium, causing adenomyosis, or activates embedded ectopic endometrial glands to grow deeper into the myometrium, resulting in deeper adenomyosis [8]. This might explain dysmenorrhea as a main clinical feature of EA failure that requires further intervention. Women with dysmenorrhea might have endometriosis and/or adenomyosis and are therefore at risk of persistent pelvic pain after endometrial ablation, which treats neither of the two underlying conditions. The presence of pre-existing dysmenorrhea is the most strongly correlated risk factor for receiving a surgical reintervention such as hysterectomy [1]. Women’s health practitioners should be aware that failure to correctly diagnose adenomyosis might result in the inadvertent use of ablation, leading to further intervention. Improved diagnostic accuracy of adenomyosis may reduce the number of women undergoing inappropriate ablation. This might require wider use of MRI, which has a higher sensitivity and specificity than ultrasound in diagnosing adenomyosis [10]. This is a small retrospective cohort study. The average follow-up of 1.6 years remains short, and the longer-term hysterectomy rate is yet to be defined by future studies. It is not known if the adenomyosis was present prior to EA or developed subsequent to EA. There is no pathological proof of the underlying pathology being treated by UAE. Future studies could be designed to use MRI to document the absence of adenomyosis prior to EA and see if there is a reduction in EA failure rate.

## Conclusions

UAE is safe and effective in managing women who failed endometrial ablation, with significant improvement in dysmenorrhea and heavy menstrual bleeding. Most women were able to avoid hysterectomy following UAE for ablation failure.

### Supplementary Information

Supplementary Material 1.

## Data Availability

Data is available for review upon request.
