# Supplementary material for: Uterine artery embolisation for adenomyosis in women who failed prior endometrial ablation
Source: CVIR Endovasc. 2024 Jul 27;7:59. doi: 10.1186/s42155-024-00471-5 (PMC11283443; doi:10.1186/s42155-024-00471-5)
Supplement: Supplementary file 1 — Supplementary Material 1. [file 42155_2024_471_MOESM1_ESM.pdf]

Appendix 1 On-line survey Part 1

Dear (first name),

Thank you for agreeing to participate in this audit program so that we can better understand the long term effectiveness of the embolisation treatment for women suffering from menstrual issues. It should not take you more than 5-10 minutes.

This on-line questionnaire is conducted on a secure database server. All the information you provided will be kept confidential and only be available to Dr Eisen Liang, his clinic staff, his research assistant and students.

Please answer the following questions:

Q1. Do you still have menstrual periods? Yes / No

Q2a. If you are in menopause, when did this happen? -______________(Month and Year)

Q2b. If menopause occurred within 6 months of the procedure, were you unhappy about this?

1. No, not unhappy

2. Yes, unhappy.

Q3. Since the procedure, how are the periods like now (or just before menopause)?

1. Lightest Ever

2. Lighter than my usual / normal

3. Back to my usual / normal

4. Still heavy

5. Heavier than before the procedure

Q4a. If you had period pain before the procedure, how is the period pain now (or just before menopause)?

0. (I did NOT have period pain before embolisation)

1. The pain is completely gone.

2. The pain is much better.

3. The pain improvement is slight.

4. The pain is still the same

5. The pain is worse than before the embolisation

Q4b. What is the pain score now (or just before menopause)?

(Imagine on a scale of 10, 0 being no pain at all, 10 being worse imaginable pain)

0 1 2 3 4 5 6 7 8 9 10

Q5. If you had bladder or pressure symptoms, how the symptoms now?

1. All gone

2. Much improved

3. Slightly improved

4. No improvement

5. Worse than before treatment

Q6. Since the embolisation, have you had any other treatments for your menstrual issues?

1. Nil

2. Medications

3. Mirena

4. Laparoscopy f

5. Hysterectomy

6. Others (Please comment)

____

Q7. In terms of the outcome of the procedure until now (or until menopause), what would you say:

1. I am very happy (or very satisfied).

2. I am happy (or satisfied).

3. I am not sure.

4. I am not happy (or not satisfied).

5. I am very unhappy (or very unsatisfied).

Q8. Did you fall pregnant, if so what happened?

1. No
2. Full term, no issues
3. Miscarried
4. Terminated / aborted
5. Premature/complicated

Q9. Is there anything else you would like to say to Dr Liang and his team?

Comments:

{Submit now and continue}

You might remember when you saw Dr Liang at the clinic; you filled in a symptom survey? We would like to see how you score now (or at the time just before your menopause).

It will take only about 3 minutes of your time.

{Begin symptom and quality of life survey}

UFS-QOL Survey

{Submit}

Thank you so much for participating in this clinical audit program. You input will help us to help other women dealing with menstrual issues.
